# “I Want to Keep the Personal Relationship With My Doctor”: Understanding Barriers to Portal Use among African Americans and Latinos

**DOI:** 10.2196/jmir.5910

**Published:** 2016-10-03

**Authors:** Courtney Rees Lyles, Jill Y Allen, Dolly Poole, Lina Tieu, Michael H Kanter, Terhilda Garrido

**Affiliations:** ^1^ University of California San Francisco San Francisco, CA United States; ^2^ Kaiser Permanente Northern California Division of Research Oakland, CA United States; ^3^ Kaiser Permanente National Market Research Oakland, CA United States; ^4^ Kaiser Permanente Hospitals, Quality and Care Delivery Excellence Oakland, CA United States; ^5^ Kaiser Permanente Southern California Permanente Group Pasadena, CA United States; ^6^ The Permanente Federation Oakland, CA United States; ^7^ Kaiser Permanente Health Information Technology Transformation and Analytics, Oakland CA, United States. Terhilda Garrido is now with Camino Advisors, LLC, Oakland, CA, United States

**Keywords:** electronic health record, African Americans, Hispanic Americans, qualitative research

## Abstract

**Background:**

Despite the widespread implementation of electronic health records (EHRs), there is growing evidence that racial/ethnic minority patients do not use portals as frequently as non-Hispanic whites to access their EHR information online. This differential portal use could be problematic for health care disparities since early evidence links portal use to better outcomes.

**Objective:**

We sought to understand specific barriers to portal use among African American and Latino patients at Kaiser Permanente, which has had a portal in place for over a decade, and broad uptake among the patient population at large.

**Methods:**

We conducted 10 focus groups with 87 participants in 2012 and 2013 among African American and Latino Kaiser Permanente members in the mid-Atlantic, Georgia, and Southern California regions. Members were eligible to participate if they were not registered for portal access. Focus groups were conducted within each racial/ethnic group, and each included individuals who were older, had a chronic disease, or were parents (as these are the three biggest users of the portal at Kaiser Permanente overall). We videotaped each focus group and transcribed the discussion for analysis. We used general inductive coding to develop themes for major barriers to portal use, overall and separately by racial/ethnic group.

**Results:**

We found that lack of support was a key barrier to initiating portal use in our sample—both in terms of technical assistance as well as the fear of the portal eroding existing personal relationships with health care providers. This held true across a range of focus groups representing a mix of age, income, health conditions, and geographic regions.

**Conclusions:**

Our study was among the first qualitative explorations of barriers to portal use among racial/ethnic subgroups. Our findings suggest that uniform adoption of portal use across diverse patient groups requires more usable and personalized websites, which may be particularly important for reducing health care disparities. This work is particularly important as all health care systems continue to offer and promote more health care features online via portals.

## Introduction

The US Health Information Technology for Economic and Clinical Health (HITECH) Act has provided more than US $25 billion in federal incentive dollars to implement electronic health records (EHRs) across health care clinics and systems across the United States. As we move forward with EHR implementation, there is increased emphasis on understanding patient needs and preferences for accessing portal websites that are linked to EHRs. This issue is particularly important given the growing evidence that patient access to and use of portals (which include viewing laboratory test results and visit summaries and allowing email communication between patients and health care providers) are linked to improved satisfaction [1] as well as better outcomes [2-4]. For example, several recent studies of systems that have had portals for over a decade (such as Kaiser Permanente) have shown that patient portal use is associated with better overall quality of care indicators [5].

Although portals are being widely implemented and may be contributing to improved health outcomes, there is evidence that they are not accessed equally across groups despite uniformly high patient interest in and enthusiasm for portals [6-8]. It is well documented that racial/ethnic minorities are significantly less likely to use portals in integrated delivery systems [9-11] as well as community-based clinics [12]. However, the differences in portal use are not fully understood and cannot be attributed to computer/Internet access alone [10]. Within the handful of studies examining adoption of portal use overall, there are several major barriers that have emerged. Some of the potential reasons previously cited for nonuse include lack of awareness [8,13], lack of sufficient computer skills [10,13,14], reduced ability to understand medical content or limited health literacy skills [8,15,16], poor usability of portal websites/interfaces [15,16], need for provider or system support [17], and concerns about security of information online [8,10].

There have been few qualitative studies to date that have specifically examined barriers to portal use by race/ethnicity, despite the documented findings of lower rates of portal use among racial/ethnic minority groups. Therefore, we designed a qualitative study of non-Hispanic African American and English-speaking Hispanic/Latino patients at Kaiser Permanente to explore this issue in depth. We hypothesized that the barriers to adoption of portal use would differ within the groups based on their interests, preferences, and concerns.

## Methods

We conducted this qualitative study at Kaiser Permanente, which has offered a version of the patient portal in various regions of the United States since the mid-2000s. Collectively, this health care system serves 10 million patients, with well over half (5.5 million) already using the online patient portal website. Specifically, we recruited Kaiser Permanente members from the Mid-Atlantic, Georgia, and Southern California regions for this study to ensure geographic and racial/ethnic diversity in the patients sampled (explained more in depth below).

### Recruitment and Focus Group Processes

We conducted 10 focus groups with Kaiser Permanente patients who were not registered for the online patient portal. The portal (also known as “kp.org”) allows patients access to several features:

(1) viewing medical history including visit summaries, immunizations, and allergies, (2) viewing laboratory results, (3) refilling medications, (4) making appointments, and (5) sending a secure message to a health care provider. All of these features were available on both a Web browser and a mobile app at the time of the study. The portal served as an alternative means to access these services, as they were often also available through in-person, mail, or phone platforms.

We limited our sample to individuals who were patients of Kaiser Permanente for at least 2 years and had visited a Kaiser Permanente facility in the past year, were English-speaking, were at least somewhat familiar with the patient portal, and used the Internet at least once a week (no other assessment of participants’ digital/computer literacy was collected). These recruitment criteria ensured that the sample was a stable group of Kaiser Permanente members who were capable of accessing the portal on their own. In particular, we understood that language barriers might be a particular barrier for Latino patients but believed that we could recruit only English speakers in this study since the portal was available only in English. The focus groups were also targeted to key groups that had health care coordination needs and therefore might be most inclined to use the portal for specific tasks.

In October 2012, we conducted six of the focus groups specifically with African American patients, holding two focus groups each in three Kaiser Permanente regions nationwide (Mid-Atlantic, Georgia, and Southern California). This included two focus groups with older adults, two focus groups with patients with chronic illness, and two focus groups with parents of young children (Table 1). Focus groups were also additionally stratified by income level (based on categories that reflected the income distributions of Kaiser Permanente patients) and age when possible to increase variation in the participants across the groups. Finally, within each focus group we ensured a mix of gender, employment status, education, and marital status during recruitment. In December 2013, we used the same process to conduct four additional focus groups with Latino patients, all conducted in Southern California (Table 2). This included two focus groups with parents and two focus groups with patients with chronic illness.

**Table 1:** African American focus groups.

	Group 1, n=9	Group 2, n=9	Group 3, n=10	Group 4, n=5	Group 5, n=10	Group 6, n=9
Location	Mid-Atlantic	Mid-Atlantic	Georgia	Georgia	Southern California	Southern California
Demographic/ Health focus	Older adults	Chronic illness	Chronic illness	Parents	Older adults	Parents
Household income, US $	Any	≤$40K or $41-80K	$41-80K or ≥$81K	≤$40K or $41-80K	Any	$41-80K or ≥$81K
Age, years	55+	35-59	35-59	30-44	60+	30-54

**Table 2:** English-speaking Latino focus groups in Southern California.

	Group A, n=10	Group B, n=9	Group C, n=8	Group D, n=8
Demographic/Health focus	Chronic illness	Parents	Chronic illness (2 or more)	Parents
Household income, US $	Any	$41-80K or ≥$81K	Any	≤$40K
Age, years	35-59	24-34	35-54	24-39
Older adults	Mix	No	Mix	No

The focus groups were led by 2 experienced moderators who were racially/ethnically concordant with the study sample. All focus groups were held in independent market research facilities and lasted approximately 2 hours. Each group consisted of 8-10 participants (with the exception of one focus group with only 5 participants), and participants received a US $100 incentive for participation. All sessions were videotaped and the conversations were later transcribed for analysis. The study was approved by the Kaiser Permanente Southern California Institutional Review Board.

The discussions focused on (1) current health status and relationship with health care providers, (2) current Internet/technology use, (3) knowledge of Kaiser Permanente and the patient portal, (4) review of the health care services and health content available online, and (5) barriers and facilitators to adoption of the patient portal. The full discussion guide is included in Multimedia Appendix 1.

### Analysis

Our qualitative analysis began with open coding of all the transcripts, focusing in on portions of the conversations that raised potential barriers and facilitators to use [18]. One member of the team (CRL) created the initial codebook, based on the discussion guide questions and a review of the previous qualitative literature documenting barriers to portal use in the general patient population. Then, at least 2 researchers read each transcript and coded using both the original codebook and open coding whenever necessary. The entire team met regularly to review the approach, edit the codes (collapsing or creating new codes as needed), and come to consensus on the themes and their interpretation as they emerged [19]. We also compared the coded segments within each theme to one another in a spreadsheet, which allowed us to identify and report on the richest information rather than quantifying the number and type of barrier categories. Overall, this process allowed for several checks on the validity of the final results by making sense of ambivalent and contradictory statements and articulating themes that were common across key informant interviews.

The primary themes presented here were fully saturated among both African Americans and Latinos and are therefore presented combined. When subsequently stratifying the analysis by race/ethnicity, we also identified one additional theme that was specific to each group.

## Results

### Sample Demographics

Overall, there were 87 individuals who attended the focus groups (Table 3). The sample was 60% female (52/87), 45% (39/87) low-income (≤US$40,000 annual household income), and 54% (47/87) aged 45 or older. In addition, 30% (26/87) of respondents had diabetes and 30% (26/87) had hypertension. The African American focus groups were slightly more female (69%, 36/52) versus Latinos (46%, 16/35), older (68%, 35/52 aged 45 or older) compared to Latinos (32%, 11/35), with a higher proportion of respondents with hypertension (40%, 21/52) versus Latinos (20%, 7/35), and a lower proportion who were low-income (37%, 19/52) compared to Latinos (57%, 20/35).

**Table 3:** Participant demographics.

		African American, n=52	Latino, n=35	Total, n=87
**Age (years), %**				
	24-39	21	57	35
	40-49	23	28	25
	50-74	56	15	40
**Gender, %**				
	Male	31	54	40
	Female	69	46	60
**Income (USD), %**				
	≤$40K	37	57	45
	$41-80	54	43	49
	≥$81K	10	0	6
**Education (highest level completed), %**				
	Less than high school	2	6	3
	High school degree	26	54	38
	Some college or 2-year college degree	46	37	43
	4-year college degree	13	3	9
	Postgraduate degree	12	0	7
**Chronic condition, %**				
	Hypertension	40	20	30
	Diabetes	23	40	30
	Asthma	17	0	10
	Cancer	4	0	2

### Technological Proficiency

Although all participants were current Internet users based on the inclusion criteria of the study, the focus group discussions among participants uncovered a mix of technological skills. The patients who were younger tended to be the most Internet-savvy, but even a substantial proportion of the older participants/chronic illness patients could perform sophisticated tasks online, including researching medical treatments and conducting banking transactions: “I’ve had health questions that I’ve gotten online to find out cures, and alternative medicine, and things like that” (African American female, focus group 5 with older adults) and “If you’re on your smartphone it’s so much easier, with one click you have everything you need” (Latina female, focus group B with parents).

However, older adults and those with chronic illness (who were also older on average) tended to make most of the comments related to limited computer proficiency: “And I tell myself, I see some seniors out there and they’re texting and they’re going online and I say if they can do that, I can do it and it isn’t that difficult. So like it’s a challenge for me” (African American female, focus group 5 with older adults).

### Major Reasons for Portal Nonuse

Our detailed coding and analytic process revealed several primary barriers to use of the patient portal, all outlined in Table 4. Four of the themes were directly linked to the need for feeling supported and/or connected, either from health care providers or from the health care system: all of which are explored in depth below.

**Table 4:** Additional themes outlining barriers to portal use.

Theme	Latino/Hispanic	No. of comments	Black/African American	No. of comments
Not technically savvy	Compared to some of these kids, and all they do is live out of their computer, I don’t. I’m barely learning. (focus group C, Male)	9	Then you’ve got 100 passwords – you’ve got one for the bank, for the school, you’ve got the ABT, and you’ve got to be remembering all of this. (focus group 1, Female)	15
			For me it’s taking the time to get into all this new technology. I’m old school. I’m not used to all that. (focus group 3, Male)	
	I’m old school. I’m very computer illiterate. Somebody at work tells me “You have to do...” I’m like “Oh, can you do it for me? I don’t know what to do.” When it comes to sitting in front of a computer, I sit in front of a computer all day, but I just do input. (focus group A, Female)		I don’t go on the Internet. I don’t look for any medical anything on the Internet. (focus group 5, Male)	
Concern portal would interfere with personal relationships	When you’re talking to a person, you can tell if that person cares about what you’re talking about. (focus group C, Male)	11	Let’s have something personal with the doctor. Everything else is automated and animated. (focus group 1, Male)	16
	[On why it’s better to get results from doctor vs online:] He [my doctor] would explain it to me more in detail, and he keeps telling me over and over, every single visit, how I'm doing this bad. (focus group C, Male)		The trust factor is really important. (Female, focus group 5)	
			That [secure messaging] is not going to sub for having him [my doctor] look at me. (focus group 1, Female)	
Prefer talking to live person	I tried to use it to find things out, but at the same time, to me I feel like I need to talk to people. I can’t deal with computers, or stuff like that, because you’re not talking to a human person that can answer to you right there and things like that. (focus group C, Female)	28	I’d rather be called in for everything good or bad because if the only time you’re calling me in, is if something is wrong, I don’t really want to go. I’d rather go in for everything. (focus group 1, Male)	34
	I just like a live person…Yes. A live person. Computers only do so much, and I like a live person just in case I have a question. Especially, like he said, you look at your results, what the hell do they mean? (focus group C, Male)		I want to hear the voice and know that they care. When you get test results, if there’s nothing wrong with you, they’ll be a letter. (focus group 4, Female)	
Need help to register	When I went to the website, I was trying to sign in to all the process but for some reason it didn’t work. I gave up, I just contacted them. (focus group B, Male)	14	I didn’t understand anything [on kp.org]. They said put your name and your zip code – it was too much. (focus group 1, Female)	11
Think existing systems are working fine	Certain people, like myself, you stick to ways that work for you. (focus group B, Female)	3	I never have a problem with them. I don’t get sick that often, so I really don’t know if it works or it doesn’t work to be online or not. I don’t know if it would be a convenience for me or not. (focus group 4, Female)	23
kp.org needs to be simple to understand/use	If you go to kp.org there is information for either view, basic information, health information but I think what it is it’s a lot of reading and it’s overwhelming. (focus group A, Female)	12	[If I saw a lab result online I didn’t understand,] I’d pick up the phone and call them asking what it means. (focus group 4, Female)	8
Concerns about security	They can hack into phones, they can steal my information. I wouldn’t use it. I only use it for music and calls. That’s it. (focus group D, Male)	3	There are hackers out to get you…We’re paranoid scared about who is looking in our stuff. (focus group 1, Female)	26

#### Concern Online Tools Would Diminish Personal Relationships With Health Care Providers

A main theme of the discussions was the need to protect or establish interpersonal relationships with health care providers. Many participants stated that they knew their health care provider was invested in them when he or she took time to talk about their health and wellness during visits or followed up with them personally via phone calls after visits: “When I do go in there with a long list of certain things going on with me, he answers. He gives me more than 15 minutes. He calls me by name” (African American female, focus group 1 with older adults).

That’s one thing I liked about my Doctor, Dr. X. He wanted to know what was happening in your life. It wasn’t just because your test was wrong or low or whatever. It’s like, let’s see what’s happening in your life, to see what’s contributing to your health problems.

Latino male, focus group C with 2 or more chronic conditions

Because of this high level of importance on the patient-provider relationship, it was clear that several participants were skeptical of portals supporting their relationships. Some even expressed concern that portals would decrease their existing quality of care or be used to replace face-to-face visits altogether: “I really don’t want to get into it [the portal]. I don’t want them to get used to me going to kp.org. I want to keep the personal relationship with my doctor” (African American male, focus group 6 with parents).

I really have concerns about the email. The doctor ratio patient, per email, if he has 500 patients and I’m just saying hypothetically. He receives 200 directly from patients, plus the nurses typing, the respond time, the time for him seeing his patients, if everybody joined this and started texting all kinds of stuff, what care are these physicians now going to provide? Seriously, it concerns me.

African American female, focus group 2 with chronic illness

These comments collectively suggest participants’ worry that portals would interfere with existing visits. This was true regardless of the status of the participant’s existing relationship with their health care provider: those with negative relationships felt the portal could block the ability to establish an interpersonal relationship, and those with positive relationships often stated that portals could threaten their personal connection.

#### Stated Preferences for In-Person Communication

Because there was such as strong emphasis on relationships with health care providers, this was naturally directly related to many stated preferences for face-to-face or phone communication. This was the case overall (ie, being the type of person who liked in-person communication throughout all aspects of life) and particularly true for health-related communication because of the importance of the discussion content: “When you have something that’s wrong with you, like diabetes, I think that’s when you would want a little bit more personal” (Latina female, focus group A with chronic illness), “I want to look at you and I want to talk to you. I want you to see me” (African American female, focus group 1 with older adults), and “I’m not a big email person and I just feel like, especially when it comes to my health, I would prefer to be face to face with my doctor” (African American female, focus group 3 with chronic illness).

However, when exploring this preference more in-depth, more nuance emerged. Some of the comments about preferring in-person communication could be additionally interpreted as a need for personal reassurance or verification. That is, these individuals did not feel as though they could comprehend the provided information sufficiently through online communication alone, or were worried they would not be sufficiently understood: “I like also to confirm when I am speaking to a live person. I like to confirm that I spoke to somebody” (Latino male, focus group D with parents) and “I can deal with the phone, but I really don’t like talking to anybody over the phone because I had a bad experience with someone on the phone, he couldn’t understand what I was saying, but when you’re in their presence it’s different” (African American male, focus group 4 with parents).

Taken together, these comments about preferring in-person communication reflected a mix of personal values and confidence in the health care system (often based on previous experiences at Kaiser or other health care institutions). Face-to-face communication was critical to assure patients that they understood health care information correctly and that the health care provider/system was not making a mistake in some way—that is, a safeguard to ensure the highest quality of care possible.

#### Portal Not Easy/Simple Enough to Use

There was also a sentiment that many participants needed more concrete support and/or technical assistance for portal use. This was the case regardless of existing computer use since all participants had to be weekly Internet users to be included in our final sample. In addition, this theme emerged in focus groups regardless of whether they predominantly comprised low-income patients, older adults, or with those with chronic illness. First, there were some general comments about trying to navigate more sophisticated websites: “I think a lot of it is just the simplicity to get into it…The simpler it is, the easier to get into it, to look at it. If it’s complex—I’m not going to look at it” (Latino male, focus group C with 2 or more chronic conditions) and “It’s just I know I can blame myself for it but I’m just like, I’m very forgetful with passwords. I know, it’s like every 3 or 4 months, I’ll probably forget my password. I know I can write it down, but most people don’t” (Latino male, focus group D with parents).

In addition, this was especially true for the portal registration process. While all participants were *not* portal users based on the inclusion criteria for the study, some had previously (but unsuccessfully) attempted to register for portal use: “When I heard about it [the portal], I went on it, and when it asked me for the password and wouldn’t give it, I said ‘Forget it. I don’t know how to do it.’” (Latina female, focus group A with chronic illness), “When I went to the website, I was trying to sign in to all the process but for some reason it didn’t work. I gave up, I just contacted them” (Latino male, focus group B with parents), and “It was too difficult when I tried it…It said to create a password. It took you through a whole bunch of stuff, and I finally got frustrated and stopped” (African American male, focus group 1 with older adults).

Because of the perceived difficulties in using the website, there were also several comments about needing more training or one-on-one support from the health care system to be able to access the portal: “Maybe Kaiser can provide some kind of guidance to help you use it, and what you can find, and how you can do things through them, and be able to get that information available, and it’s easier” (Latina female, focus group C with 2 or more chronic conditions).

I’m going to do it [use the portal] only when I can do it for myself. I don’t need anybody to do it for me. When I can walk in, fire up the computer, and do everything that I’m supposed to do for myself…This is what I’m saying. When I can just walk in and do it for myself. I don’t have to ask for help, I’d consider that then.

African American male, focus group 1 with older adults

#### Portal Content Is Often Too Complex

Finally, some participants noted that, beyond basic functionality, the website content could also be challenging. This was strongly related to the ability to interpret the medical information provided: “If you go to kp.org, there is information for either view, basic information, health information. But I think what it is it’s a lot of reading and it’s overwhelming” (Latina female, focus group A with chronic illness) and “Is it user friendly? Is it terms that we can understand, laymen’s terms?” (African American female, focus group 2 with chronic illness).

If I have a question because sometimes when you get these lab results with all these medical terms, they don’t break it down into what they really mean, so sometimes I have to call my doctor and say, “What is this about, what does it mean, what do these numbers represent?”

African American male, focus group 5 with older adults

### Specific Themes for African Americans and Latinos

We sought to explore whether there were differences in barriers to portal use for African Americans compared to Latinos; however, most of the themes from the focus groups were similar in both groups. There were two exceptions to note. First, African American respondents appeared to be more concerned about the security/privacy of their information online: “If you can crack the Pentagon and the White House and all that, first of all, they didn’t even ask if they could put my medical records online” (female, focus group 5 with older adults).

Because my medical history and my medical business is my business, and when you have hackers and all kinds of foolishness going on in the Internet that may go into somebody else’s spam folder and that’s my medical history. I’m not comfortable with that.

female, focus group 3 with chronic illness

Second, Latino respondents were more consistent in their comments about using the mobile phone app to access the site, as they felt strongly this would be more usable than a website: “A website might be much more complicated but the app is broken down to very simple. It’s on your phone, there’s not so much you can do” (female, focus group B with parents) and “If I had an app on my phone, then I could just know...I don’t have to look for it through the internet, I could just click on it and there it is, and just sign in, and make it easier” (male, focus group C with 2 or more chronic conditions)

## Discussion

### Principal Findings

Our study was among the first to specifically examine barriers to portal use among African American and Latino patients. Among a national sample of Kaiser Permanente patients, we found that lack of support was a primary barrier in using the online patient portal. More specifically, participants wanted both additional technical assistance in using the portal and expressed worry that the portal could undermine their existing in-person relationships with their health care providers. This held true across a range of focus groups representing a mix of age, income, health conditions, and geographic region. These findings are particularly important because of the well-documented lower portal use within these two racial/ethnic groups across health care systems.

### Comparison to Previous Work

Our findings are consistent with previous literature on this topic, which suggests that the categories of barriers for racial/ethnic minority groups may not be substantially different from those seen in the overall patient population. For example, as stated above, previous studies have also found that connections with health care providers and comfort with using computers were challenges in using portal websites [13,14,17]. However, our findings go deeper on many of the themes than the previous literature was able to do. For example, participants expressed not just a desire for closer relationships with their provider, but the need to protect in-person visits from the “threat” of online communication, indicating anxiety about portal use replacing the interpersonal aspects of their existing relationships. In addition, our findings indicate a multifaceted perspective on preferences for in-person communication that might be more prevalent than in the dominant culture. This may include stronger cultural value placed on face-to-face communication or utilizing in-person communication as a coping strategy to ensure that the patient is receiving the highest quality of care possible. It is also important to note in our other national market research (results available among request), mainstream Kaiser Permanente portal users reported feeling very differently from the participants in this study: that the portal strengthened their relationships with health care providers.

Furthermore, it is clear from our study that basics of website usability, such as simplicity in design and content delivery, are also barriers to portal use. A large proportion of individuals in our study, even those who used the computer or mobile phone for other tasks, reported that the portal seemed too complex to access. This is consistent with previous evidence that documented lower usability of portal websites among racial/ethnic minority patients as well as those with limited health literacy [20,21]. Moreover, the need for health literacy training or support was also evident, such as the lack of confidence in being able to independently interpret medical content presented on a portal without one-on-one assistance, which is also consistent with other health technology work [22,23]. While literacy and socioeconomic status can trend together, the health literacy needs noted in this study were not confined to those from lower socioeconomic status alone. Because portals display patient-specific medical content from the EHR that is meant for provider use, there is ongoing work that needs to be done to ensure that portals are accessible and usable.

With respect to the subgroup-specific theme related to security and privacy of information online, we found that African Americans expressed more concerns about portals compared to Latinos. This might be related to age [24], as the Latino focus groups were slightly younger than the African American focus groups, or broader cultural mistrust of the health care system based in the unique historical context for this racial/ethnic group, such as the unethical treatment in the Tuskegee Syphilis Experiment [25,26]. However, it is important to note that in the overall Kaiser Permanente population, older adults have the highest rate of portal registration and use. This suggests that patient education/communication about the security measures taken to protect portal websites (including the limits to that security) should be well developed, potentially targeted to older patient groups first, and should be clear about the specific contexts of use (eg, who is viewing the information and for what purpose [27]).

### Limitations

Our study has some limitations to note. First of all, our sample included only Kaiser Permanente patients, and so the barriers to portal use in this integrated delivery system may not be comparable to other health care settings or insurance types. However, it is important to note that Kaiser Permanente cares for patients from all sociodemographic groups and multiple private and public insurer types, making it an extremely heterogeneous patient population overall. In addition, while we did include African American patients from across the country, the Latino patients were all residents of Southern California. We did not include patients from other races/ethnicities, which would allow for more comparisons between racial/ethnic groups, including direct comparisons to white and East Asian patients at Kaiser Permanente who use the portal at the highest rates in this health care setting. Finally, we focused only on portal nonusers and therefore developed a more detailed understanding of barriers (rather than facilitators) to use.

### Conclusions

Moving forward, our findings have implications for clinical practice. One of the key messages of this study is that personal relationships can substantially support and encourage use. This is particularly important with respect to having clear expectations about what types of communication are best delivered through in-person versus online channels, which might help to assure patients that interpersonal aspects of communication will be prioritized and preserved. In addition, this study suggests that additional support or training for digital and health literacy skills might enhance portal use for some patient subgroups.

At a system level, there are a number of targeted strategies already underway to address some additional barriers identified in this study. For example, the Kaiser Permanente portal registration process continues to be enhanced to simplify and streamline the experience for patients. In addition, a TRUSTe security seal was added to the homepage of the portal to assure visitors of their privacy when they interact with the Kaiser Permanente website. Also, the marketing messages for patients are being refreshed to promote the availability of the mobile app and to reinforce that the portal is a convenient way for patients to have more personalized contact with their health care providers. Future studies should examine the effectiveness of these system-level efforts in ultimately increasing portal use rates across racial/ethnic groups.

In conclusion, our study identified the need for personalized and technical support to encourage African American and Latino patients to use portals for their health care management. As the United States continues to shift toward patient engagement and patient-centered care, it is critical to ensure that health technologies like portals are usable for all patient groups. Portals are a platform through which many health care systems plan to integrate additional mobile health technologies, such as uploading patient-generated sensor or mobile phone app data into the EHR. As health care systems move to collecting and sending more electronic data to and from patients, it is critical that this process addresses broad barriers to use and reduces the possibilities of exacerbating existing health care disparities.

## Appendix

Multimedia Appendix 1 Focus Group Discussion Guide.

## Abbreviations

EHR: electronic health record
