# Supplementary material for: “I Want to Keep the Personal Relationship With My Doctor”: Understanding Barriers to Portal Use among African Americans and Latinos
Source: J Med Internet Res. 2016 Oct 3;18(10):e263. doi: 10.2196/jmir.5910 (PMC5067358; doi:10.2196/jmir.5910)
Supplement: Multimedia Appendix 1 [file jmir_v18i10e263_app1.pdf]

APPENDIX

Focus Group Discussion Guide

Before we get started, let's go around and briefly introduce ourselves. Tell us your name, describe your family/home situation (spouse, significant others, children), and how you spend most of your time.

Today we're going to talk about you/your family's health and how you manage you/your family's health. I want you to understand that this is not meant to be judgmental in any way. I would appreciate your honesty in sharing your experiences.

I. Current Health Situation (15 minutes)

I want to get a quick picture of where you are health wise.

A. How would you describe your current health status? What about your family's health status?
   - (PROBE FOR GENERAL CONDITION,CHRONIC HEALTH ISSUES, OR DAILY CHALLENGES SUCH AS WEIGHT OR ALLERGIES.)

B. How important is it to you to be able to manage your health concerns for yourself and your family?

C. Do you have any specific health goals? What are they?

D. How do you "manage you/your family's health" towards those health goals? What do you do specifically?

   (PROBE FOR BEHAVIOR, DIET, EXERCISE, MEDICAL TREATMENTS, AVAILABLE TOOLS AND RESOURCES FOR INFORMATION.)

E. How are your current efforts working for you?

   (PROBE FOR SUCCESS STORIES AND

   SETBACKS.)

F. I want you to think of the number #1 health issue that you are currently dealing with. Identify it and tell me what you want or need to better manage that issue.

   (PROBE FOR INFORMATION, BEHAVIOR, DIET, EXERCISE, MEDICAL TREATMENTS, SUPPORT.)

G. In general, what would you want or need that would help you better manage your health?

   (PROBE FOR COUNSELLING, AVAILABLE TOOLS, COMMUNITY RESOURCES, AND SOURCES FOR INFORMATION.)

H. What resources do you rely upon to manage your health?

   (PROBE FOR DOCTOR, HEALTH PLAN, CLINIC, HOSPITAL, FAMILY, COMMUNITY GROUPS, SUPPORT GROUPS, AND WEBSITES)
   - Which resources do you trust the most or consider to be the

most credible? Why?

I. Do you view “managing your health” as a strictly personal concern or a broader community concern? Please explain.

(IF NOT MENTIONED, PROBE FOR IMPACT ON AFRICAN-AMERICAN COMMUNITY AT LARGE.)

II. Relationship With Doctor (15 Minutes)

A. You mentioned your doctor as a resource. How many doctors are you currently seeing? Which one do you consider to be your primary care doctor or the one you see most often?

- What type of relationship do you have with your doctor?
- How long have you been with your current doctor?
- How did you select your doctor? What was important to you?

(PROBE FOR ETHNICITY, COMMUNICATION, TYPE OF CARE/RESPONSIVENESS, TRUST ISSUES, REFERRALS, LOCATION, CONVENIENCE)

B. Outside of an office visit, how do you prefer to communicate with your doctor – in person, telephone call, letter, e-mail, text? Why?

(PROBE FOR ACCESS, CONVENIENCE, COMFORT, GOOD/BAD NEWS SITUATIONS)

C. What is the best way to communicate general health information to you? Personal health information to you? Why?

(PROBE FOR IN PERSON, TELEPHONE CALL, LETTER, E-MAIL, TEXT)

III. INTERNET USAGE (10 Minutes)

A. We’ve been talking about ways to get information to you. Do you use the Internet? How often – daily, twice a week, weekly?

- Does anyone in your household help you when you use the computer?
- Is this your office computer, home computer, or a public computer?
- What type of Internet service is available to you – dial up, cable, FIOS, satellite, wireless?

B. Typically what are you doing on the Internet – searching for information, shopping, managing your bank account, paying bills, planning trips, etc.?

C. Which websites do you visit most often? (PROBE FOR HEALTH, DIET, NUTRITION, EXERCISE) What are you looking for when you visit these sites? What type of information?

IV. Kaiser Permanente (10 minutes)

A. It’s my understanding that everyone here is a member of Kaiser Permanente. What role does Kaiser Permanente play in helping you manage your health?

B. How do you use Kaiser Permanente to help manage your health concerns? (IF “DON’T”, PROBE WHY NOT.)

- What resources are available to you through Kaiser Permanente for managing your health?

(PROBE ADVICE NURSE, BROCHURES, HEALTH EDUCATION CLASSES,WEIGHT WATCHERS, INFORMATION, ANY SPECIAL PROGRAMS, WEBSITE, VISITS TO EDUCATION HEALTH CENTERS)

C. Overall, which resources are the most helpful, the easiest to use and understand for you? Why?

V. Awareness of Kp.org (20 minutes)

A. Someone mentioned Kp.org. How many of you have visited the Kp.org website?

- (FOR THOSE WHO HAVE NOT) Why haven't you visited Kp.org?
- How did you hear about Kp.org? (PROBE FOR:)
    - Mailing from Kaiser Permanente
    - Kaiser Permanente doctor or other health care provider
    - Family member or friend
    - Radio or television advertisement
    - Workplace or employer
    - Internet
    - Other

(IF DOCTOR,MEDICAL STAFF, OR FRIEND MENTIONED)

- What did they tell you about Kp.org?
- Has anyone had any prior experience with Kp.org? How was it?
- What were you doing/what were you looking for?
- What can you tell me about Kp.org?
- What do you think is the purpose of Kp.org?

B. It is my understanding that you have not registered at Kp.org. Why not? What are your primary reasons for not registering at Kp.org?

(PROBE FOR TIME, CONVENIENCE, EASY ACCESS, PERCEIVED VALUE, TECHNOLOGY CONCERNS – ACCESS, USE OF PASSWORDS, SECURITY – SYSTEMIC CONCERNS, PRIVACY CONCERNS – INTERPERSONAL TRUST CONCERNS, LACK OF INFORMATION, HEALTH LITERACY -UNDERSTANDING OF MEDICAL INFORMATION.)

C. Has your doctor or any medical staff spoken to you about Kp.org? What did they say?

- How important would it be to hear about Kp.org from your doctor or medical staff? Why?

D. What do you want and need to know about Kp.org to better understand its purpose and how to use Kp.org?

E. What do you need to know that would encourage you to register for Kp.org?

F. What specifically would motivate you to register for Kp.org?

VI. Presentation of Specific Features Of Kp.org (30 minutes)

I want to share with you some information about specific features that you will have access to once you have registered for Kp.org.

A. PRESENT EACH DESCRIPTIVE PARAGRAPH.

- Email your doctor – Send secure, routine, messages to your doctor and get a response within two business days.
- View most lab test results – Get most lab test results as soon as available – many on the same day.
- Refill prescriptions – Order your prescription refills and have most of them mailed to your home with – free shipping.
- Schedule, cancel, or review routine appointments – Schedule routine appointments and check past office visit information for recommended follow-up steps.
- View recent immunizations and allergies and more – Review names and dates of your immunizations, a list of allergies, and your eligibility and benefits information.
- Act for a family member – Access portions of your family members' health information and e-mail their doctors using our secure online features.

AFTER EACH, ASK:

B. How important is this feature/information to you?

- How likely are you to use this feature?
- What would it enable you to do now that you could not before? How would you use this?
- What are the advantages of using this feature? Any disadvantages?

C. AFTER PRESENTATION OF ALL,

- Based on what you've seen, which feature has the most appeal to you? Why?
- Which has the most value to you? Why?
- Can you see yourself registering at and using Kp.org? Why/Why not?

- (FOR THOSE WHO DON'T) What would motivate you to register at Kp.org? What do you need to know?
- Would you use Kp.org to manage you/your family's health?
- Of all of the things you have seen and heard about Kp.org, what is the most important thing for you? What has the most value?
- Would you encourage other members of your family to register and use Kp.org? Why/Why not?
- Do you still have about concerns about registering at Kp.org.? What are they?
- What other questions do you have about Kp.org?

VII. Information Resources (5 minutes)

A. What would be the best way to communicate and engage with you about your health?

- Through a family member
- Direct Mail
- On-line – E-mail
- Kaiser Permanente website
- Doctor, medical staff
- Workplace or Employer
- Church/Social Organizations
- Senior Centers
- Community events (Festivals, health fairs, community meetings)
- Social Media (Facebook, Twitter)
- Smart Phone/IPhone Mobile Apps

Closing

Do you have any other questions about the Kp.org?

Thank you.
